# Association of GLP-1 Receptor Agonist Use with Thyroid Outcomes in Adults with Type 2 Diabetes

**DOI:** 10.7150/ijms.141099

**Published:** 2026-10-02

**Authors:** Ching-Chung Hsiao, Ming-Hsien Tsai, Jian-Yu Jhu, Ya-Fang Liu, Chieh-Li Yen, Jia-Jin Chen, Pei-Chi Chen

**Affiliations:** 1 Department of Nephrology, New Taipei Municipal Tu Cheng Hospital, New Taipei City, Taiwan; 2 College of Medicine, Chang Gung University, Taoyuan, Taiwan; 3 Kidney Research Center, Linkou Chang Gung Memorial Hospital, Taoyuan, Taiwan; 4 Division of Nephrology, Department of Internal Medicine, Shin-Kong Wu Ho-Su Memorial Hospital, Taipei, Taiwan.; 5 Department of Medicine, Fu Jen Catholic University, New Taipei City, Taiwan; 6 Data Analytics Group, Department of Digital Medicine, Shin-Kong Wu Ho-Su Memorial Hospital, Taipei, Taiwan; 7 Division of Endocrinology and Metabolism, Department of Internal Medicine, Shin-Kong Wu Ho-Su Memorial Hospital, Taipei, Taiwan; 8 Department of Nephrology, Linkou Chang Gung Memorial Hospital, Taoyuan, Taiwan

**Keywords:** glucagon-like peptide-1 receptor agonist, Type 2 diabetes mellitus, autoimmune thyroiditis, thyroid disorders, dipeptidyl peptidase-4 inhibitor, real-world evidence

## Abstract

**Background:**

Glucagon-like peptide-1 receptor agonists (GLP-1 RAs) are widely used to treat type 2 diabetes mellitus, but their associations with thyroid disorders remain uncertain. We evaluated whether initiating a GLP-1 RA, compared with initiating a dipeptidyl peptidase-4 inhibitor (DPP-4i), was associated with newly recorded autoimmune thyroiditis and other thyroid outcomes.

**Methods:**

This new-user, active-comparator cohort study emulated a target trial using the TriNetX Collaborative Network. Adults with type 2 diabetes who initiated a GLP-1 RA or DPP-4i between January 1, 2018, and December 31, 2022, and had no prior use of either drug class were eligible. Patients with pre-existing thyroid disease, any neoplasm, immunosuppressant or immunostimulant use, transplantation, or end-stage kidney disease or dialysis were excluded. Propensity score matching yielded 107,912 patients per group. The primary outcome was newly recorded autoimmune thyroiditis; secondary outcomes were nontoxic uninodular goiter, nontoxic multinodular goiter, and thyroid malignancy. Cox models were used to estimate hazard ratios (HRs) and 95% confidence intervals (CIs), with false discovery rate adjustment. Follow-up extended from 1 day after treatment initiation to 4 years.

**Results:**

Autoimmune thyroiditis occurred in 524 GLP-1 RA users (0.49%) and 417 DPP-4i users (0.39%). GLP-1 RA initiation was associated with a higher risk of autoimmune thyroiditis (HR, 1.26; 95% CI, 1.11-1.43; FDR-adjusted *P* = 0.002). Modestly higher risks were also observed for nontoxic uninodular goiter (HR, 1.07; 95% CI, 1.01-1.13) and nontoxic multinodular goiter (HR, 1.11; 95% CI, 1.03-1.19). The risk of thyroid malignancy did not differ significantly (HR, 0.93; 95% CI, 0.77-1.12). Findings were generally consistent across sensitivity and subgroup analyses, although heterogeneity was observed across body mass index strata.

**Conclusion:**

Among adults with type 2 diabetes, GLP-1 RA initiation was associated with small increases in newly recorded autoimmune thyroiditis and benign nodular thyroid disease, but not with thyroid malignancy. Given the small absolute differences and the potential for residual confounding and surveillance bias, these findings require validation in future studies.

## Introduction

Glucagon-like peptide-1 receptor agonists (GLP-1 RAs) have become central to the management of type 2 diabetes mellitus (T2DM) because of their potent glucose-lowering effects, weight-reduction benefits, and established cardiovascular and kidney protection 1-3. However, their potential thyroid effects remain a concern. In rodent studies, GLP-1 RAs induced medullary thyroid carcinoma (MTC) 4, 5. This effect has been attributed to activation of GLP-1 receptors on thyroid C cells, which promotes cellular proliferation and increases calcitonin levels. This preclinical signal led to regulatory warnings. Under US Food and Drug Administration guidance, GLP-1 RAs are contraindicated in individuals with a personal or family history of MTC or multiple endocrine neoplasia type 2.

The relevance of these findings to humans remains uncertain because human thyroid C cells express GLP-1 receptors at substantially lower levels than rodent thyroid C cells 6, 7. Clinical evidence regarding thyroid carcinogenesis in GLP-1 RA users is inconsistent. European pharmacovigilance data have shown disproportionately high reporting of thyroid cancer, including medullary thyroid cancer, among patients treated with GLP-1 RAs 8. However, large population-based cohort studies and meta-analyses of randomized controlled trials have not found a statistically significant increase in overall thyroid cancer risk, including MTC, among GLP-1 RA users compared with users of other antidiabetic agents 9-12. Further research is needed to clarify this potential association.

GLP-1 RAs may also affect other aspects of thyroid function, particularly by reducing serum TSH levels, although their effects on free thyroid hormones, including free T3 and T4, appear limited. Studies of exenatide and liraglutide in patients with diabetes or obesity have reported reduced TSH levels without significant changes in free T3 or T4, and one exenatide study also found reduced thyroid volume without changes in thyroid autoantibodies or nodule size 13-15. One possible mechanism is central inhibition at the hypothalamic level, where GLP-1Rs interact with TRH-producing neurons and affect the hypothalamic-pituitary-thyroid (HPT) axis 16. Weight loss may also contribute because obesity has been associated with elevated TSH in some studies 17. However, available studies have generally not shown clinically meaningful changes in circulating thyroid hormone concentrations 18.

Overall, the effects of GLP-1 RAs on thyroid disease remain incompletely understood. Previous safety concerns have focused mainly on thyroid malignancy, whereas autoimmune thyroiditis has received less attention. We selected autoimmune thyroiditis as the primary outcome because it is a clinically relevant immune-mediated thyroid condition with plausible links to GLP-1 receptor-mediated immunomodulation. As the use of GLP-1 RAs expands among patients with type 2 diabetes and obesity, it is important to determine whether exposure is associated with incident autoimmune thyroiditis. We therefore used a new-user, active-comparator target trial emulation to compare the risk of autoimmune thyroiditis between GLP-1 RA and DPP-4 inhibitor users. We also evaluated benign thyroid nodular disease and thyroid malignancy as secondary outcomes.

## Materials and Methods

### Data Source

TriNetX is a federated real-world data platform that connects healthcare organizations with researchers. Its continuously updated, anonymized electronic health record and claims data allow researchers to identify patient cohorts, assess study feasibility, and perform statistical analyses. The platform supports cohort identification, outcome analysis, and real-world study design 19. Its specialized healthcare networks also facilitate research on long-term health trends and rare diseases.

### Ethics Statement

The study protocol complied with the International Council for Harmonisation Good Clinical Practice guidelines, the Declaration of Helsinki 20, and relevant laws governing noninterventional and observational studies. TriNetX provides users with deidentified data and shares only aggregated results and statistical summaries. The Ethics Review Board of Shin Kong Wu Ho-Su Memorial Hospital approved this study (Institutional Review Board number: 20250104R) and waived informed consent because the data were deidentified. The study was reported in accordance with the Strengthening the Reporting of Observational Studies in Epidemiology (STROBE) guideline for cohort studies.

### Study Design and Population

This retrospective cohort study used a new-user, active-comparator, intention-to-treat design within a target trial emulation framework. It was conducted using the TriNetX US Collaborative Network, a large real-world data platform containing more than 118 million patient records. Target trial emulation was used to approximate the design of a randomized controlled trial and make the intended trial framework explicit 21. Dipeptidyl peptidase-4 inhibitors (DPP-4is) were selected as the active comparator because they share an incretin-based mechanism with GLP-1 RAs and are commonly used as second-line antidiabetic agents 22. We identified 4,538,091 adults (aged ≥18 years) who had at least three clinical encounters and a diagnosis of T2DM between January 1, 2018, and December 31, 2022. The index date was the date of the first prescription for the assigned study medication. Patients were then classified by antihyperglycemic treatment exposure: (1) new GLP-1 RA users with no prior use of either GLP-1 RAs or DPP-4is before the index date (n = 342,173) and (2) new DPP-4i users with no prior use of either drug class before the index date (n = 245,366). To reduce confounding, we excluded patients with conditions that could influence thyroid function or the study outcomes. These conditions included diagnosed thyroid disorders, immunosuppressant or immunostimulant use, any neoplasm, transplantation, and end-stage kidney disease or dialysis. After these exclusions, 187,424 GLP-1 RA users and 142,188 DPP-4i users remained eligible.

Propensity score matching (PSM) was performed using a 1:1 nearest-neighbor algorithm without replacement to balance baseline covariates and reduce confounding, resulting in two matched cohorts of equal size.

### Baseline Covariates

Baseline variables were assessed during the two years before the index date. Demographic variables included age at index, body mass index, systolic and diastolic blood pressure, sex, and race (White, African American, Asian, and other). Lifestyle factors included alcohol-related disorders, nicotine dependence, and tobacco use. Socioeconomic factors included potential health hazards related to socioeconomic and psychosocial circumstances, employment and unemployment issues, and housing and economic problems. Comorbidities included acute kidney failure, chronic kidney disease, atrial fibrillation and flutter, cerebrovascular diseases, dyslipidemia, liver cirrhosis, gout, heart failure, hypertensive diseases, ischemic heart disease, chronic obstructive pulmonary disease, peripheral vascular disease, mental, behavioral, or neurodevelopmental disorders, and inflammatory diseases of the central nervous system. Medications included insulin, biguanides, sodium-glucose cotransporter 2 inhibitors, sulfonylureas, thiazolidinediones, beta-blockers, renin-angiotensin system blockers, calcium channel blockers, diuretics, lipid-modifying agents, antiepileptics, antineoplastic and immunomodulating agents, and antihistamines. Laboratory variables included estimated glomerular filtration rate, urine microalbumin-to-creatinine ratio, high-density lipoprotein cholesterol, low-density lipoprotein cholesterol, triglycerides, hemoglobin, hemoglobin A1c (HbA1c), alanine aminotransferase, albumin, aspartate aminotransferase, calcium, phosphate, potassium, and sodium. The relevant diagnostic codes and laboratory data missingness rates are listed in Method S1.

### Study Outcomes

The primary outcome was incident autoimmune thyroiditis. Secondary outcomes were thyroid-related structural conditions, including solitary thyroid nodules, multinodular goiter, and malignant thyroid neoplasm. Outcomes were identified using prespecified diagnostic codes detailed in Method S1 to ensure transparency and reproducibility. Because thyroid antibody results and imaging data were not consistently available, outcomes were ascertained from diagnostic codes among patients without recorded thyroid disease before treatment initiation. Patients were followed for outcomes from 1 day to 4 years after the index date, with observation continuing through June 20, 2025. Follow-up ended at the occurrence of the outcome of interest, death, or loss to follow-up, whichever occurred first.

### Sensitivity Analyses

We included positive and negative outcome controls to evaluate the validity of our findings. Major adverse cardiovascular events (MACEs) and major adverse kidney events (MAKEs) were selected as positive controls because GLP-1 RAs have established cardiovascular 2 and kidney benefits 3 in patients with T2DM. Respiratory infection was included as an exploratory control outcome based on evidence suggesting a possible association with reduced respiratory illness 23. These outcomes allowed us to assess whether the study design could reproduce expected associations 2, 3, 23. Ketoacidosis, aortic aneurysm or dissection, and otitis media were selected as negative controls because they are not expected to be associated with GLP-1 RAs or DPP-4is; these outcomes helped assess whether the study design generated spurious associations. We also performed three sensitivity analyses. First, we used multiple PSM models with stepwise adjustment for confounders to better isolate treatment effects. Second, we evaluated risks across different follow-up periods to address potential violations of the proportional hazards assumption and assess the stability of the results over time. Finally, we extended the exposure lag window to 3 months to reduce potential reverse causation and the detection of pre-existing but previously unrecognized disease. To assess surveillance bias, we compared TSH and free T4 testing, thyroid or neck ultrasound, and outpatient visits within 1 and 4 years after the index date between the matched cohorts, using standardized mean differences rather than P values given the large sample size, and estimated hazard ratios for any recorded thyroid disorder within the same periods. The relevant diagnostic codes are listed in Method S1.

### Statistical Analysis

Categorical variables are reported as counts and percentages, and continuous variables as means and standard deviations (SDs). We used the PSM procedure available in the TriNetX platform to create comparable groups of GLP-1 RA and DPP-4i users and reduce confounding from differences in baseline characteristics. Standardized mean differences (SMDs) were used to assess the balance of demographic, clinical, and laboratory variables; an SMD of less than 0.1 indicated a small between-group difference 24, 25. Details of the PSM procedure are provided in Method S2.

Kaplan-Meier curves and log-rank tests were used to estimate event-free probabilities. Follow-up began after the index date and continued for up to 4 years. Associations between GLP-1 RA and DPP-4i use and the study outcomes were assessed using Cox proportional hazards models, with hazard ratios (HRs) and 95% confidence intervals (CIs) reported. Proportional hazards assumptions were evaluated using the generalized Schoenfeld approach in TriNetX. When these assumptions were violated, HRs were estimated within discrete time periods. We tested four hypotheses with an expected false discovery rate of 0.05. P values were adjusted using the Benjamini-Hochberg false discovery rate (FDR) procedure.

We calculated E-values to assess the potential influence of unmeasured confounding. The E-value is the minimum strength of association that an unmeasured confounder would need to have with both the exposure and outcome, conditional on the measured covariates, to fully explain the observed effect. Larger E-values indicate greater robustness to unmeasured confounding 26. Absolute effect measures, including absolute risk increase (ARI) and number needed to harm (NNH), were calculated from Kaplan-Meier estimates to aid interpretation of the clinical relevance of the observed associations 27. For subgroup analyses, each subgroup was re-matched using PSM based on baseline variables. Each stratum was defined as a separate cohort and matched independently; therefore, the numbers of matched patients and events across strata do not sum to those of the primary analysis, and strata defined by BMI, eGFR, or HbA1c included only patients with a recorded baseline value. Because interaction testing was unavailable in TriNetX, subgroup heterogeneity was assessed by comparing the stratum-specific hazard ratios with Cochran's Q test 28.

Missing data were not imputed. Subgroup heterogeneity tests, E-values, false discovery rate adjustment, and other derived statistics were calculated from the exported TriNetX results; propensity score matching and survival analyses were conducted on the TriNetX platform, with statistical significance defined as P < 0.05. Figures were generated using R version 4.3.0 (R Foundation for Statistical Computing, Vienna, Austria) with the Forestploter and ggplot2 packages.

## Results

### Baseline Characteristics

Cohort selection is shown in Figure 1, and the specification of the emulated target trial is summarized in Table S1. Before matching, the GLP-1 RA (n = 187,424) and DPP-4i (n = 142,188) cohorts differed substantially (Table 1). GLP-1 RA users were younger (55.2 vs. 61.8 years), had a higher BMI (36.6 vs. 31.8 kg/m^2^), and were less often male (48.3% vs. 53.0%). They also had lower prevalences of chronic kidney disease (9.5% vs. 15.2%), atrial fibrillation (4.1% vs. 6.8%), heart failure (5.7% vs. 8.2%), and ischemic heart disease (11.9% vs. 16.4%). The GLP-1 RA cohort had a higher eGFR (82.4 vs. 75.1 mL/min/1.73 m^2^) and hemoglobin level (13.6 vs. 13.0 g/dL). GLP-1 RA users were more likely to receive insulin (39.7% vs. 33.9%) and SGLT2 inhibitors (17.9% vs. 13.1%) but less likely to receive biguanides (53.6% vs. 60.4%) and sulfonylureas (18.2% vs. 27.5%).

After propensity score matching, each group included 107,912 patients. Baseline characteristics were well balanced, with all standardized mean differences below 0.1. Balance diagnostics are shown in the Love plot (Figure S1). Follow-up durations were also comparable between the matched cohorts (Figure S2). Mean follow-up was 1043.5 ± 438.6 days in the GLP-1 RA group and 1059.4 ± 481.9 days in the DPP-4i group. Median follow-up was 1144 days (interquartile range [IQR], 662) and 1287 days (IQR, 726), respectively.

### Association Between Autoimmune Thyroiditis and GLP-1 RA Use

Kaplan-Meier curves (Figure 2) showed a significantly lower event-free probability for autoimmune thyroiditis among patients treated with GLP-1 RAs than among those treated with DPP-4is, with early and persistent separation of the curves (log-rank P < 0.001). The GLP-1 RA group also had a higher risk of autoimmune thyroiditis than the DPP-4i group (0.49% vs. 0.39%; HR, 1.26; 95% CI, 1.11-1.43). This association remained significant after FDR adjustment (Table 2).

Over four years, GLP-1 RA use was associated with a higher absolute risk of autoimmune thyroiditis than DPP-4i use. The ARI increased from 0.04% in year 1 to 0.14% in year 4, while the NNH decreased from 2394 to 733. These findings indicate a small but increasing ARI over time (Table S2).

### Association Between Secondary Thyroid Outcomes and GLP-1 RA Use

For the secondary outcomes (Table 2), the risks of solitary thyroid nodules (HR, 1.07; 95% CI, 1.01-1.13) and multinodular goiter (HR, 1.11; 95% CI, 1.03-1.19) were modestly higher in the GLP-1 RA group. Both associations remained significant after FDR adjustment. The risk of thyroid malignancy did not differ significantly between the treatment groups.

Kaplan-Meier curves for the secondary outcomes (Figure S3) showed lower event-free probabilities for solitary thyroid nodules and multinodular goiter over four years among patients with T2DM treated with GLP-1 receptor agonists than among those treated with DPP-4 inhibitors. Both differences were statistically significant (log-rank P < 0.05).

### Sensitivity Analyses

In the positive outcome control analyses, GLP-1 RA use was associated with significantly lower risks of MACE, MAKE, and respiratory infection (all P < 0.05; Table S3). In the negative outcome control analyses, the risks of ketoacidosis, aortic aneurysm or dissection, and otitis media did not differ significantly between groups (HRs close to 1; all P > 0.05). The findings were consistent across sensitivity analyses that used different PSM models (Table S4), follow-up durations (Table S5), and a 3-month lag window to account for potential latency in outcome ascertainment (Table S6). Thyroid evaluation and thyroid diagnoses after treatment initiation are summarized in Table S7. Within 1 year, 14.8% of GLP-1 RA users and 12.4% of DPP-4i users had TSH testing, and 1.1% and 0.9% underwent thyroid ultrasound (standardized mean differences, 0.07 and 0.02). Over 4 years, the corresponding proportions were 30.1% and 25.4% for TSH testing and 3.0% and 2.6% for ultrasound, and the standardized mean difference for the number of outpatient visits was 0.13. A thyroid disorder diagnosis was recorded in 6.2% of GLP-1 RA users and 6.0% of DPP-4i users within 1 year (HR, 1.02; 95% CI, 0.99-1.06) and in 11.4% and 10.9%, respectively, within 4 years (HR, 1.02; 95% CI, 0.99-1.05).

### Subgroup Analyses

The subgroup forest plot (Figure 3) showed that the association between GLP-1 RA use and autoimmune thyroiditis, compared with DPP-4i use, was generally consistent across clinically relevant strata. Most point estimates indicated a higher risk in the GLP-1 RA group, with little evidence of effect modification. Results were broadly similar across strata defined by sex, age, race, kidney function, glycemic control, nicotine dependence, cardiovascular comorbidities, chronic kidney disease, dyslipidemia, and mental disorders; most heterogeneity P values were nonsignificant. Heterogeneity was observed across BMI categories (heterogeneity P = 0.040), with a stronger association among patients with BMI <30 kg/m^2^ (HR, 1.53; 95% CI, 1.12-2.08) than among those with BMI ≥30 kg/m^2^ (HR, 1.02; 95% CI, 0.82-1.28). No other subgroup showed significant effect modification. Numerically stronger associations were observed among patients aged ≥65 years (HR, 1.39; 95% CI, 1.13-1.72) and those with chronic kidney disease (HR, 1.57; 95% CI, 1.09-2.27).

## Discussion

In this large propensity score-matched cohort of adults with T2DM, GLP-1 RA initiation, compared with DPP-4i initiation, was associated with a statistically significant but small absolute increase in newly recorded autoimmune thyroiditis and modest increases in benign thyroid nodular disease. The risk of thyroid malignancy did not differ significantly between the treatment groups. These findings were generally consistent across sensitivity and subgroup analyses. Nevertheless, the small absolute risk differences and the potential for residual confounding, outcome misclassification, and surveillance bias require cautious interpretation.

The higher incidence of newly recorded autoimmune thyroiditis among GLP-1 RA users was a key finding, although a direct pathway linking GLP-1 receptor activation to thyroid-specific autoimmunity remains unproven. Autoimmune thyroiditis involves loss of tolerance to thyroid peroxidase and thyroglobulin, autoantibody production, and dysregulation of Th1/Th17, Th2, and regulatory T-cell responses. Because activated T cells express GLP-1 receptors, pharmacologic stimulation could theoretically alter this immune balance or unmask subclinical disease in susceptible individuals 29-31. GLP-1 RAs may also influence the hypothalamic-pituitary axis and cytokine profiles 32, which raises the possibility of an indirect effect on thyroid autoimmunity. However, direct evidence that GLP-1 RAs increase thyroid autoantibody production or activate thyroid-specific immune responses is lacking. Human data on GLP-1 RAs and autoimmune thyroid disease remain limited. Our findings are consistent with the hypothesis that GLP-1 receptor signaling may influence thyroid autoimmunity in susceptible individuals, but observational data cannot establish causality or the underlying mechanisms.

We also observed a modest increase in benign nodular thyroid disease, including solitary nodules and multinodular goiter, among GLP-1 RA users. The biological and clinical relevance of this finding remains uncertain. GLP-1 signaling may influence thyroid cell proliferation or vascularization, although direct evidence in human thyroid tissue is limited. Because thyroid nodules can have clinical implications, especially in patients with underlying autoimmune disease, this signal warrants further study.

We found no significant difference in the incidence of thyroid malignancy between GLP-1 RA and DPP-4i users. This finding is consistent with recent large observational studies, including a Nordic registry-based analysis that found no association between GLP-1 RA exposure and thyroid cancer risk after accounting for latency and treatment duration 33. Concern about GLP-1 RAs and thyroid cancer stems largely from rodent studies in which chronic GLP-1 RA administration caused C-cell hyperplasia and medullary thyroid carcinoma (MTC) 4. Species-specific differences in GLP-1 receptor expression on thyroid C cells may explain why this signal has not translated to humans. Human C cells have minimal GLP-1 receptor expression, and clinical trials have not shown a consistent increase in serum calcitonin or C-cell pathology 34, 35.

Although some studies have reported GLP-1 receptor expression in papillary thyroid carcinoma (PTC) tissue, functional studies have not shown proliferative effects of GLP-1 RAs in these cells 36. Our findings therefore provide reassuring real-world evidence that GLP-1 RAs were not associated with an increased risk of thyroid malignancy. They support the continued use of these agents in appropriate patients with T2DM while maintaining standard precautions for those with a history of MTC or MEN2 37.

Subgroup analyses showed a stronger association between GLP-1 RA initiation and autoimmune thyroiditis among patients with BMI <30 kg/m² (HR, 1.53; 95% CI, 1.12-2.08) than among those with BMI ≥30 kg/m² (HR, 1.02; 95% CI, 0.82-1.28; heterogeneity P = 0.040). Obesity is associated with chronic low-grade inflammation and altered Th17-regulatory T-cell homeostasis, which could modify or attenuate the additional immunologic effects of GLP-1 receptor activation 38, 39. Selection may also contribute: because GLP-1 RAs are usually prescribed for patients with higher body weight, adults with a BMI <30 kg/m² who initiate a GLP-1 RA may be enriched for latent autoimmune diabetes in adults or other autoimmune predisposition, which would raise their baseline risk of thyroid autoimmunity independently of treatment. Within each BMI stratum, the treatment groups were separately matched, including on insulin use and HbA1c. Associations were also numerically stronger among adults aged ≥65 years and patients with CKD, potentially reflecting immune senescence, impaired immune tolerance, and the chronic inflammatory milieu associated with kidney dysfunction 40, 41.

This study has several strengths. We used a large, multi-institutional real-world dataset and rigorous PSM to obtain well-balanced cohorts with broad representation. We evaluated autoimmune, structural, and malignant thyroid outcomes, providing a broader perspective than studies focused only on thyroid cancer. Multiple sensitivity analyses, including positive and negative outcome controls, supported the consistency of the findings, and subgroup analyses explored variation across clinically relevant patient strata. The study also has several limitations. First, residual confounding may persist despite careful matching, as in any observational study. Extra-thyroidal autoimmune diseases were not included in the propensity score model. Before matching, type 1 diabetes codes were recorded in 2.32% of GLP-1 RA users and 1.22% of DPP-4i users, each of the other 13 conditions in less than 1%, and all SMDs were below 0.1 (Table S8). In a bias analysis treating these conditions as a single unmeasured confounder 42, the hazard ratio would remain 1.21 (95% CI 1.06-1.37) even if they increased the risk of autoimmune thyroiditis fivefold. Second, we could not account for potentially important variables, including iodine status, thyroperoxidase (TPO) antibody titers, thyroid ultrasound findings, and detailed family history. Third, outcomes were defined using diagnostic codes, which may have resulted in misclassification or underreporting. Antibody results, thyroid function tests, and ultrasound findings were not consistently available to validate autoimmune thyroiditis or nodular disease. If under-recording was similar in both groups, the hazard ratios would be largely unaffected, and nondifferential false-positive coding would bias them toward the null; an overestimate would instead require more complete recording, or more false-positive coding, among GLP-1 RA users, as discussed below for surveillance bias. The same code-based ascertainment reproduced the expected results for the positive and negative control outcomes (Table S3). Fourth, we did not stratify the analyses by individual GLP-1 RA agent or dose, although their pharmacodynamic effects on the thyroid may differ. Fifth, follow-up may not have been long enough to fully assess malignancies with a long latency. Sixth, multiple subgroup comparisons were performed without adjustment for multiplicity. Finally, surveillance bias is an important concern because GLP-1 RA users may have more frequent follow-up for metabolic disease, weight management, or adverse-effect monitoring, which could increase the detection of autoimmune thyroiditis or thyroid nodules. GLP-1 RA users had modestly more thyroid testing than DPP-4i users (TSH testing within 1 year, 14.8% vs. 12.4%; thyroid ultrasound, 1.1% vs. 0.9%; Table S7), whereas the risk of any recorded thyroid disorder did not differ significantly (HR, 1.02; 95% CI, 0.99-1.06), the hazard ratio for autoimmune thyroiditis was similar across follow-up windows (Table S5) and after a 3-month lag (Table S6), and thyroid cancer was not increased.

## Conclusion

Among patients with T2DM, GLP-1 RA use was associated with a modestly higher risk of autoimmune thyroiditis and benign thyroid nodular disease, but not thyroid malignancy. Clinicians may consider individualized thyroid monitoring for selected patients, particularly those with autoimmune risk factors or older age. Prospective studies are needed to confirm these findings and clarify the underlying mechanisms.

## Supplementary Material

Supplementary figures and tables.

## Figures and Tables

**Figure 1:**
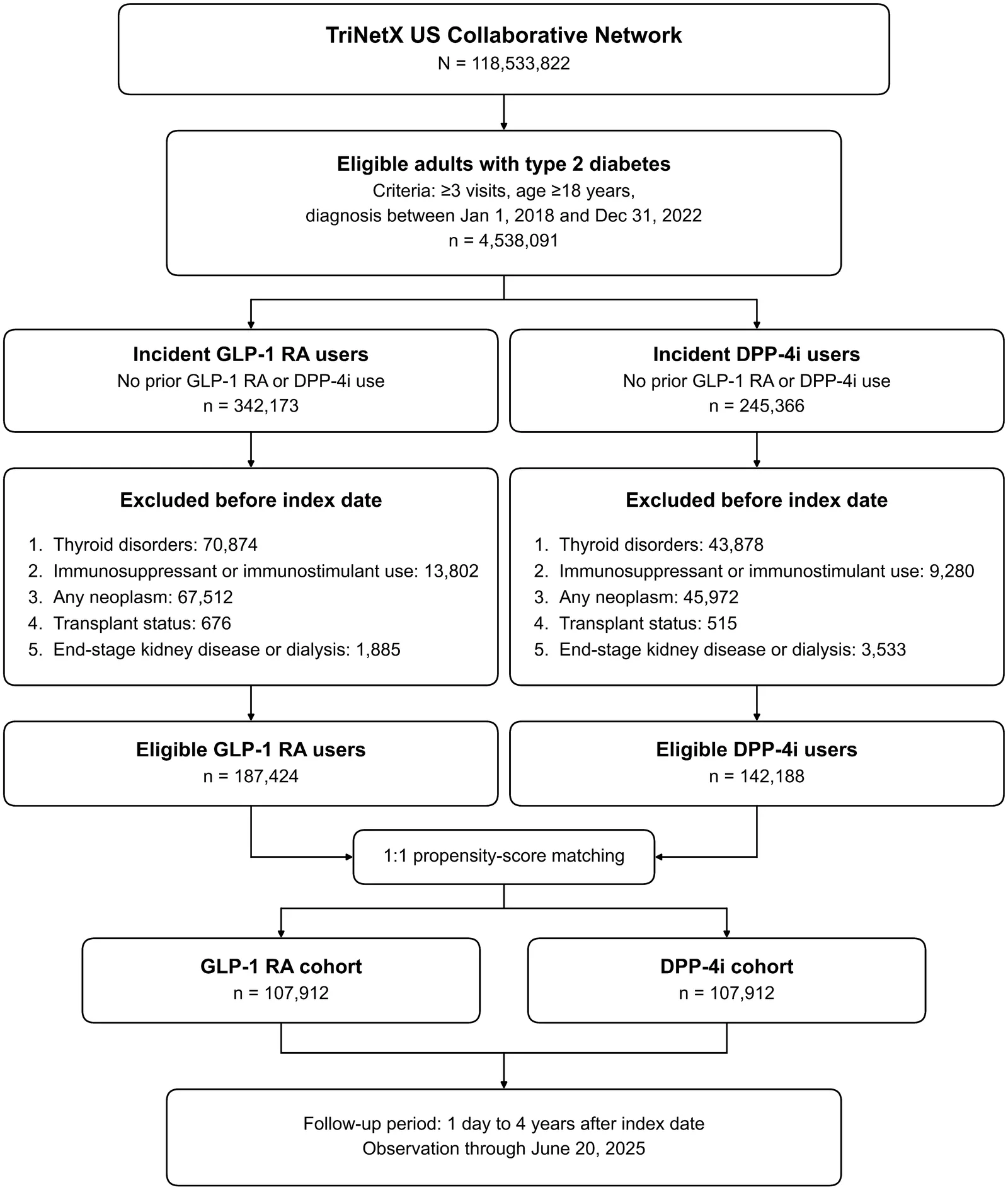
Flow diagram of patient enrollment. The index date was defined as the date of GLP-1 RA or DPP-4i initiation. Abbreviations: GLP-1 RA, glucagon-like peptide-1 receptor agonist; DPP-4i, dipeptidyl peptidase-4 inhibitor.

**Figure 2:**
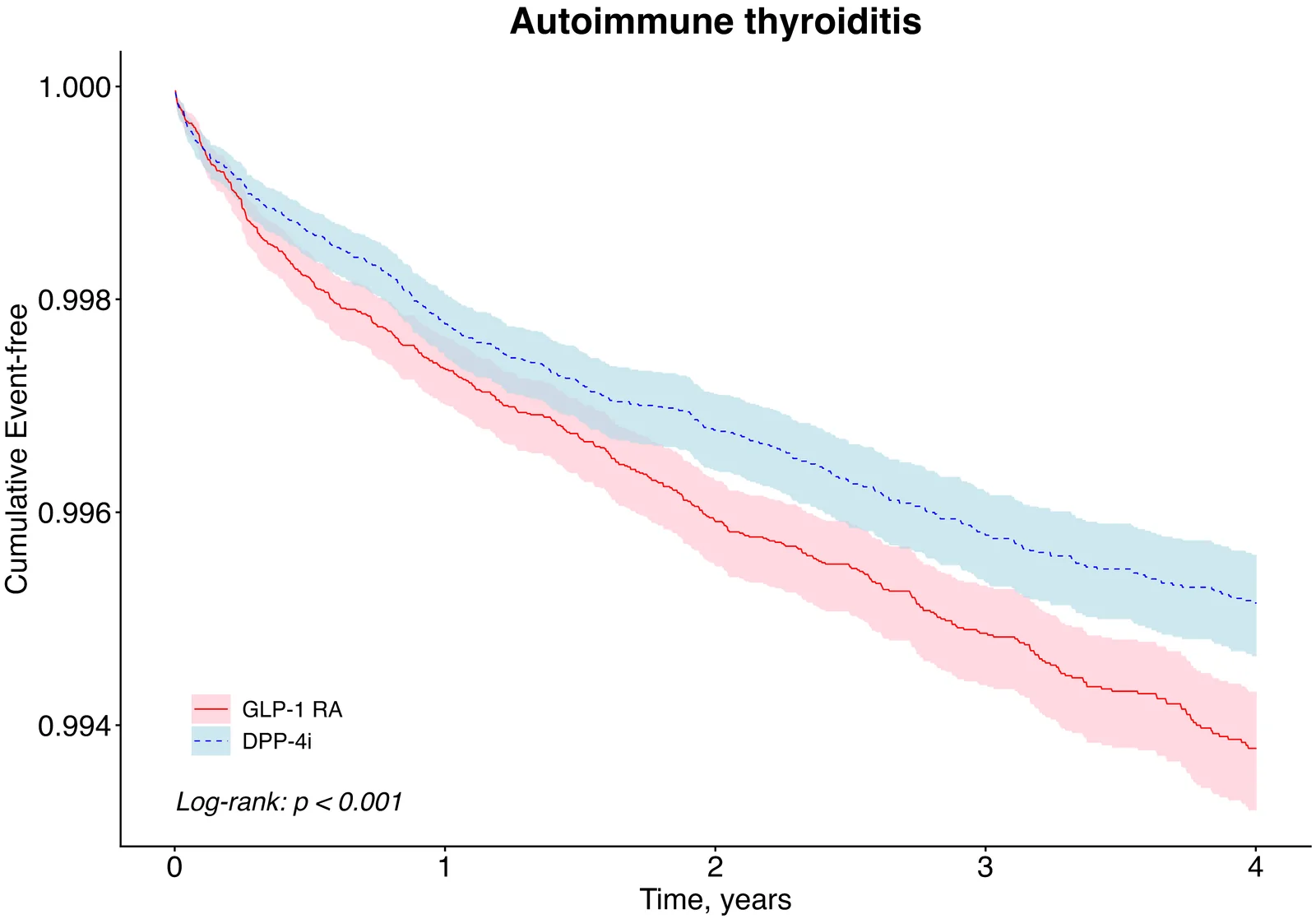
Kaplan-Meier curves for autoimmune thyroiditis-free survival. Abbreviations: GLP-1 RA, glucagon-like peptide-1 receptor agonist; DPP-4i, dipeptidyl peptidase-4 inhibitor.

**Figure 3:**
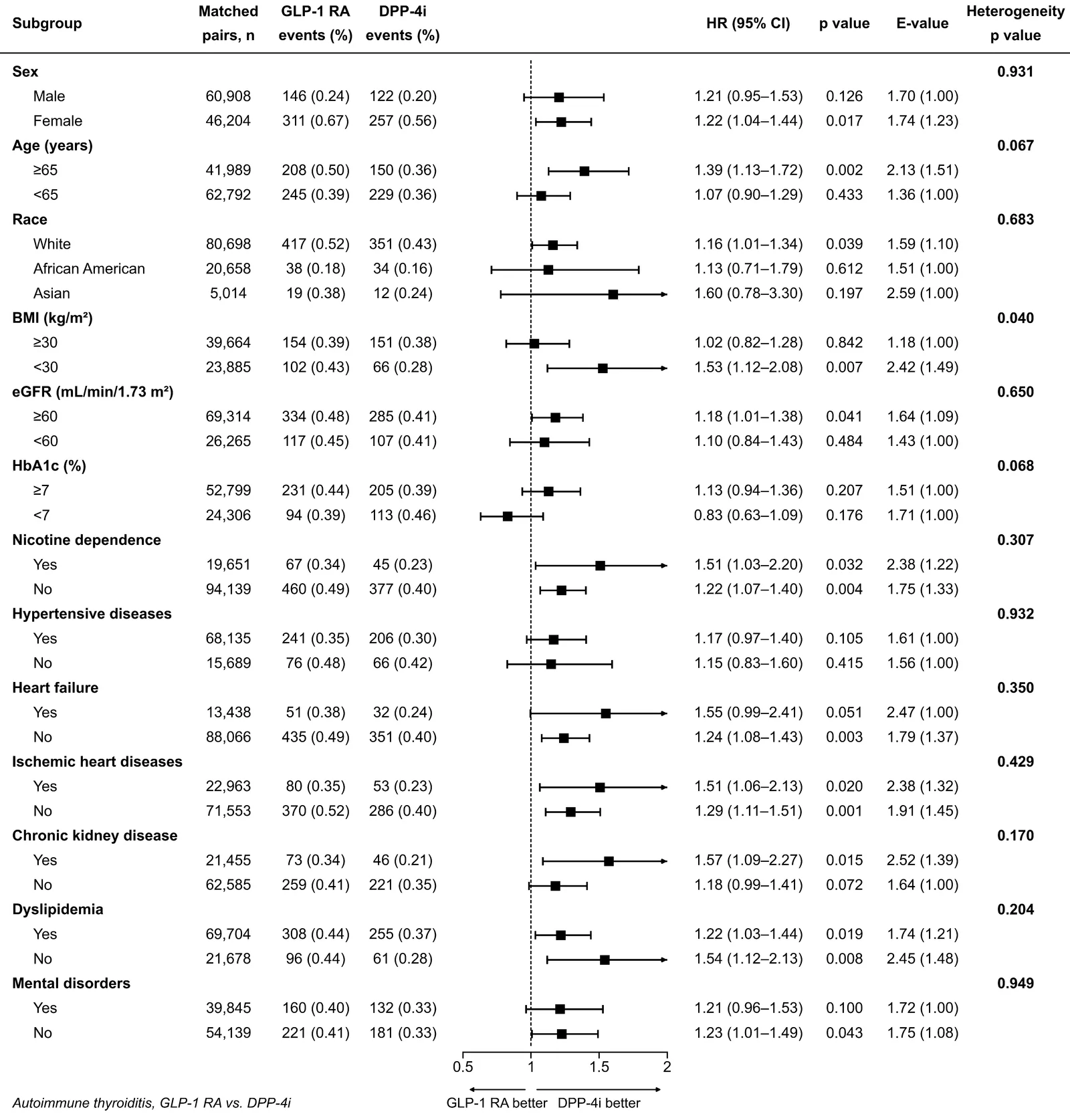
Subgroup analysis of autoimmune thyroiditis. Autoimmune thyroiditis risk was compared between patients receiving GLP-1 RAs and those receiving DPP-4is. Each stratum was propensity score matched separately, and the number of matched pairs is shown for each stratum. Abbreviations: GLP-1 RA, glucagon-like peptide-1 receptor agonist; DPP-4i, dipeptidyl peptidase-4 inhibitor; BMI, body mass index; HbA1c, hemoglobin A1c; HR, hazard ratio; CI, confidence interval.

**Table 1:** Baseline Patient Characteristics.

Variables	Before matching			After matching		
	GLP-1 RA	DPP-4i	SMD	GLP-1 RA	DPP-4i	SMD
	(n = 187,424)	(n = 142,188)		(n = 107,912)	(n = 107,912)	
**Age at index (years)**, mean ± SD	55.2 ± 13.1	61.8 ± 12.9	0.515	59.2 ± 12.0	59.2 ± 12.7	0.002
BMI (kg/m²)	36.6 ± 8.4	31.8 ± 7.6	0.594	34.1 ± 7.8	33.4 ± 7.6	0.090
Systolic blood pressure (mmHg)	130.8 ± 17.5	130.2 ± 20.2	0.030	131.1 ± 18.6	130.3 ± 19.3	0.042
Diastolic blood pressure (mmHg)	77.4 ± 11.3	74.1 ± 12.6	0.277	76.3 ± 11.7	75.3 ± 12.2	0.082
**Sex,** Male**, n (%)**	90,554 (48.3)	75,330 (53.0)	0.093	55,822 (51.7)	55,741 (51.7)	0.002
**Race, n (%)**						
White people	111,458 (59.5)	78,564 (55.3)	0.085	62,916 (58.3)	62,964 (58.3)	0.001
African American	35,692 (19.0)	25,079 (17.6)	0.036	19,495 (18.1)	19,654 (18.2)	0.004
Asian	7,183 (3.8)	11,474 (8.1)	0.180	5,679 (5.3)	5,509 (5.1)	0.007
Other race	7,414 (4.0)	6,996 (4.9)	0.047	4,732 (4.4)	4,704 (4.4)	0.001
**Lifestyles, n (%)**						
Alcohol-related disorders	2,017 (1.1)	2,264 (1.6)	0.045	1,423 (1.3)	1,430 (1.3)	0.001
Nicotine dependence	12,837 (6.8)	11,487 (8.1)	0.047	7,976 (7.4)	7,928 (7.3)	0.002
Tobacco use	4,854 (2.6)	3,672 (2.6)	<0.001	2,808 (2.6)	2,842 (2.6)	0.002
**Economic circumstances, n (%)**						
Persons with potential health hazards related to socioeconomic and psychosocial circumstances	2,218 (1.2)	1,836 (1.3)	0.010	1,299 (1.2)	1,281 (1.2)	0.002
Problems related to employment and unemployment	299 (0.2)	192 (0.1)	0.006	176 (0.2)	162 (0.2)	0.003
Problems related to housing and economic circumstances	911 (0.5)	851 (0.6)	0.015	554 (0.5)	551 (0.5)	<0.001
**Comorbidity, *n* (%)**						
Acute kidney failure and chronic kidney disease	17,832 (9.5)	21,576 (15.2)	0.173	12,978 (12.0)	13,005 (12.1)	0.001
Atrial fibrillation and flutter	7,623 (4.1)	9,677 (6.8)	0.121	5,706 (5.3)	5,610 (5.2)	0.004
Cerebrovascular diseases	7,119 (3.8)	9,421 (6.6)	0.127	5,440 (5.0)	5,418 (5.0)	0.001
Dyslipidemia	93,212 (49.7)	68,559 (48.2)	0.030	52,310 (48.5)	52,094 (48.3)	0.004
Liver cirrhosis	1,596 (0.9)	1,623 (1.1)	0.029	1,089 (1.0)	1,063 (1.0)	0.002
Gout	3,712 (2.0)	3,381 (2.4)	0.027	2,343 (2.2)	2,339 (2.2)	<0.001
Heart failure	10,599 (5.7)	11,657 (8.2)	0.100	7,292 (6.8)	7,302 (6.8)	<0.001
Hypertensive diseases	102,494 (54.7)	81,521 (57.3)	0.053	59,887 (55.5)	59,728 (55.3)	0.003
Ischemic heart diseases	22,216 (11.9)	23,288 (16.4)	0.130	15,244 (14.1)	15,198 (14.1)	0.001
Other chronic obstructive pulmonary disease	6,850 (3.7)	7,644 (5.4)	0.083	4,903 (4.5)	4,862 (4.5)	0.002
Peripheral vascular disease, unspecified	4,314 (2.3)	4,046 (2.8)	0.034	2,786 (2.6)	2,788 (2.6)	<0.001
Mental, behavioral, and neurodevelopmental disorders	48,919 (26.1)	34,228 (24.1)	0.047	26,110 (24.2)	25,928 (24.0)	0.004
Inflammatory diseases of the central nervous system	185 (0.1)	242 (0.2)	0.020	135 (0.1)	133 (0.1)	0.001
**Medication, *n* (%)**						
Insulin	74,315 (39.7)	48,219 (33.9)	0.119	37,715 (34.9)	37,537 (34.8)	0.003
Biguanides	100,418 (53.6)	85,888 (60.4)	0.138	63,213 (58.6)	62,762 (58.2)	0.008
SGLT2i	33,508 (17.9)	18,691 (13.1)	0.131	16,742 (15.5)	16,415 (15.2)	0.008
Sulfonylureas	34,022 (18.2)	39,167 (27.5)	0.225	25,425 (23.6)	25,059 (23.2)	0.008
Thiazolidinediones	7,413 (4.0)	6,621 (4.7)	0.035	4,865 (4.5)	4,818 (4.5)	0.002
Alpha-glucosidase inhibitors	332 (0.2)	528 (0.4)	0.037	266 (0.2)	260 (0.2)	0.001
Beta-blockers	48,887 (26.1)	49,976 (35.1)	0.198	33,478 (31.0)	33,272 (30.8)	0.004
Renin-angiotensin system blockade	87,767 (46.8)	75,974 (53.4)	0.132	55,189 (51.1)	55,006 (51.0)	0.003
Calcium channel blockers	35,902 (19.2)	36,720 (25.8)	0.160	24,392 (22.6)	24,290 (22.5)	0.002
Diuretics	54,460 (29.1)	45,831 (32.2)	0.069	33,514 (31.1)	33,440 (31.0)	0.001
Lipid-modifying agents	98,598 (52.6)	87,662 (61.7)	0.184	63,234 (58.6)	62,858 (58.2)	0.007
Antiepileptics	37,472 (20.0)	29,205 (20.5)	0.014	22,186 (20.6)	22,121 (20.5)	0.001
Antineoplastic and immunomodulating agents	9,673 (5.2)	6,175 (4.3)	0.038	4,761 (4.4)	4,778 (4.4)	0.001
Antihistamines	31,803 (17.0)	25,262 (17.8)	0.021	18,898 (17.5)	18,786 (17.4)	0.003
Amiodarone	1,814 (1.0)	2,581 (1.8)	0.072	1,451 (1.3)	1,462 (1.4)	0.001
Estrogens	4,376 (2.3)	1,858 (1.3)	0.077	1,686 (1.6)	1,663 (1.5)	0.002
**Laboratory, mean ± SD**						
eGFR (mL/min/1.73 m²)	82.4 ± 28.8	75.1 ± 30.8	0.246	77.9 ± 28.4	78.3 ± 30.2	0.013
UACR (mg/g)	151.6 ± 531.4	151.0 ± 524.6	0.001	171.2 ± 588.2	135.1 ± 475.5	0.067
HDL-C (mg/dL)	42.4 ± 13.4	43.0 ± 13.7	0.047	42.8 ± 13.8	42.6 ± 13.4	0.014
LDL-C (mg/dL)	94.8 ± 39.1	92.5 ± 40.3	0.059	91.8 ± 39.1	94.0 ± 40.4	0.056
Triglycerides (mg/dL)	204.1 ± 208.5	191.0 ± 202.4	0.064	196.5 ± 193.2	198.1 ± 217.0	0.008
Hemoglobin (g/dL)	13.6 ± 2.0	13.0 ± 2.2	0.293	13.4 ± 2.0	13.3 ± 2.1	0.059
Hemoglobin A1c (%)	8.7 ± 2.2	8.5 ± 2.1	0.102	8.6 ± 2.1	8.6 ± 2.1	0.006
Alanine aminotransferase (U/L)	32.9 ± 37.3	31.7 ± 55.3	0.025	31.5 ± 36.6	32.6 ± 54.5	0.024
Albumin (g/dL)	4.1 ± 0.5	3.9 ± 0.6	0.226	4.0 ± 0.5	4.0 ± 0.6	0.056
Aspartate aminotransferase (U/L)	26.8 ± 44.2	28.1 ± 55.2	0.026	26.7 ± 40.6	27.6 ± 49.9	0.019
Calcium (mg/dL)	9.4 ± 0.6	9.3 ± 0.7	0.155	9.4 ± 0.6	9.3 ± 0.6	0.066
Phosphate (mg/dL)	3.5 ± 0.8	3.5 ± 0.9	0.037	3.5 ± 0.8	3.5 ± 0.9	0.025
Potassium (mmol/L)	4.3 ± 0.5	4.2 ± 0.5	0.026	4.3 ± 0.5	4.2 ± 0.5	0.064
Sodium (mmol/L)	138.0 ± 3.0	137.9 ± 3.4	0.034	138.0 ± 3.1	137.8 ± 3.3	0.058

Data are mean ± SD or n (%), as indicated for each variable. Laboratory values are the mean of all measurements for each patient during the 2-year baseline window; the missingness rate for each laboratory value after matching is reported in Method S1. After matching, UACR was available for 12.0% of GLP-1 RA users and 11.8% of DPP-4i users. An SMD below 0.1 indicates a negligible difference between the groups. Abbreviations: BMI, body mass index; DPP-4i, dipeptidyl peptidase-4 inhibitor; eGFR, estimated glomerular filtration rate; GLP-1 RA, glucagon-like peptide-1 receptor agonist; HDL-C, high-density lipoprotein cholesterol; LDL-C, low-density lipoprotein cholesterol; SD, standard deviation; SGLT2i, sodium-glucose cotransporter 2 inhibitor; SMD, standardized mean difference; UACR, urine albumin-to-creatinine ratio.

**Table 2:** Thyroid-Related Clinical Outcomes After Matching.

Outcomes	GLP-1 RA users (n = 107,912)		DPP-4i users (n = 107,912)		GLP-1 RA versus DPP-4i users			
	Events (n)	Risk (%)	Events (n)	Risk (%)	HR (95% CI)	E-value^#^	Raw *p*-value	FDR-adjusted *p-*value
**Primary outcome**								
Autoimmune thyroiditis	524	0.49	417	0.39	1.26 (1.11-1.43)	1.83 (1.45)	<0.001	0.002
**Secondary outcomes**								
Nontoxic uninodular goiter	2,226	2.06	2,111	1.96	1.07 (1.01-1.13)	1.33 (1.07)	0.037	0.049
Nontoxic multinodular goiter	1,601	1.48	1,455	1.35	1.11 (1.03-1.19)	1.46 (1.22)	0.004	0.007
Malignant neoplasm of thyroid gland	209	0.19	225	0.21	0.93 (0.77-1.12)	1.37 (1.00)	0.438	0.438

#E-values indicate the minimum strength of association that an unmeasured confounder would need to have with both treatment and outcome, beyond the measured covariates balanced by propensity score matching, to fully explain away the observed association; they are not adjusted estimates. E-values were calculated from unrounded estimates on the risk-ratio scale. For estimates below 1.00, the inverse of the estimate was used. The E-value for the confidence interval was calculated using the confidence limit closest to the null value. If the 95% CI included 1.00, the CI-limit E-value was set to 1.00. Risk is the proportion of patients in each matched cohort with the outcome during 4 years of follow-up. Hazard ratios are from Cox proportional hazards models, raw P values from log-rank tests, and FDR-adjusted P values from the Benjamini-Hochberg procedure applied to the four outcomes. Abbreviations: CI, confidence interval; DPP-4i, dipeptidyl peptidase-4 inhibitor; FDR, false discovery rate; GLP-1 RA, glucagon-like peptide-1 receptor agonist; HR, hazard ratio.

## Data Availability

The data used in this study were obtained from the TriNetX Research Network under a data use agreement and are not publicly available. The authors cannot share individual-level patient data because of contractual restrictions and privacy protections. Researchers seeking access to similar data may apply directly to TriNetX, subject to institutional agreements, licensing requirements, and applicable regulatory approvals.

## Abbreviations

GLP-1 RA: glucagon-like peptide-1 receptor agonist
US: United States
DPP-4i: dipeptidyl peptidase-4 inhibitor
HR: hazard ratio
CI: confidence interval
T2DM: type 2 diabetes mellitus
MTC: medullary thyroid carcinoma
TSH: thyroid-stimulating hormone
T3: triiodothyronine
T4: thyroxine
GLP-1R: glucagon-like peptide-1 receptor
TRH: thyrotropin-releasing hormone
HPT axis: hypothalamic-pituitary-thyroid axis
STROBE: Strengthening the Reporting of Observational Studies in Epidemiology
PSM: propensity score matching
HbA1c: hemoglobin A1c
MACE: major adverse cardiovascular event
MAKE: major adverse kidney event
SD: standard deviation
SMD: standardized mean difference
FDR: false discovery rate
ARI: absolute risk increase
NNH: number needed to harm
BMI: body mass index
eGFR: estimated glomerular filtration rate
SGLT2i: sodium-glucose cotransporter 2 inhibitor
IQR: interquartile range
PTC: papillary thyroid carcinoma
MEN2: multiple endocrine neoplasia type 2
TPO: thyroperoxidase
UACR: urine albumin-to-creatinine ratio
HDL-C: high-density lipoprotein cholesterol
LDL-C: low-density lipoprotein cholesterol
