# Supplementary material for: Association of GLP-1 Receptor Agonist Use with Thyroid Outcomes in Adults with Type 2 Diabetes
Source: Int J Med Sci. 2026 Oct 2;23(11):3494–505. doi: 10.7150/ijms.141099 (PMC13685433; doi:10.7150/ijms.141099)
Supplement: Supplementary file 1 [file ijmsv23p3494s1.pdf]

Supplementary Online Content

Method S1. Codes for cohort definitions, outcomes, and baseline covariates....2
1. Cohort definition....2
2. Outcome definitions....2
3. Baseline parameters....4
Method S2. Details of propensity score matching on the TriNetX platform....7
Figure S1. Love plot....12
Figure S2. Distribution of follow-up time....13
Figure S3. Kaplan–Meier plots for secondary outcomes....14
Table S1. Target trial emulation....15
Table S2. Annual absolute risk metrics and clinical impact measures....18
Table S3. Positive and negative control outcomes after matching....19
Table S4. Models for confounding adjustment after matching....20
Table S5. Sensitivity analysis by follow-up duration after matching....21
Table S6. Sensitivity analysis using a 3-month exposure lag window....22
Table S7. Thyroid testing, thyroid ultrasound, outpatient visits, and thyroid diagnoses after treatment initiation in the matched cohorts....23
Table S8. Extra-thyroidal autoimmune diseases before propensity score matching and bias analysis for autoimmune thyroiditis....24

Method S1. Codes for cohort definitions, outcomes, and baseline covariates

1. Cohort definition

Item	Code	Name
**Inclusion criteria**		
visit	TNX:Visit	Visit
diagnosis	UMLS:ICD10CM:E11	Type 2 diabetes mellitus (at least 18 years old at event)
medication	NLM:ATC:A10BJ	Glucagon-like peptide-1 (GLP-1) analogues
medication	NLM:ATC:A10BH	Dipeptidyl peptidase-4 (DPP-4) inhibitors
**Exclusion criteria**		
diagnosis	UMLS:ICD10CM:C00-D49	Neoplasms
diagnosis	UMLS:ICD10CM:Z94	Transplanted organ and tissue status
diagnosis	UMLS:ICD10CM:N18.6	End stage renal disease
diagnosis	UMLS:CPT:1012740	Dialysis services and procedures
diagnosis	UMLS:CPT:90945	Dialysis procedure other than hemodialysis (e.g., peritoneal dialysis, hemofiltration, or other continuous renal replacement therapies), with a single evaluation by a physician or other qualified health care professional
diagnosis	UMLS:ICD10CM:E00-E07	Disorders of thyroid gland
medication	NLM:ATC:L04	Immunosuppressants
medication	NLM:ATC:L03	Immunostimulants

2. Outcome definitions

Outcomes	Item	Code	Name
**Autoimmune Thyroiditis**	Diagnosis	UMLS:ICD10CM:E06.3	Autoimmune thyroiditis
**Nontoxic single thyroid nodule**	Diagnosis	UMLS:ICD10CM:E04.1	Nontoxic single thyroid nodule
**Nontoxic multinodular goiter**	Diagnosis	UMLS:ICD10CM:E04.2	Nontoxic multinodular goiter

Outcomes	Item	Code	Name
**Malignant neoplasm of thyroid gland**	Diagnosis	UMLS:ICD10CM:C73	Malignant neoplasm of thyroid gland
**MACE**	Demographics	Deceased	Deceased
	Diagnosis	UMLS:ICD10CM:I21	Acute myocardial infarction
	Diagnosis	UMLS:ICD10CM:I63	Cerebral infarction
	Diagnosis	UMLS:ICD10CM:I62	Other and unspecified nontraumatic intracranial hemorrhage
	Diagnosis	UMLS:ICD10CM:I46	Cardiac arrest
**MAKE**	Diagnosis	UMLS:ICD10CM:N17	Acute kidney failure
	Diagnosis	UMLS:ICD10CM:N18.6	End stage renal disease
	Procedure	UMLS:CPT:1012740	Dialysis Services and Procedures
	Procedure	UMLS:CPT:90945	Dialysis procedure other than hemodialysis (e.g., peritoneal dialysis, hemofiltration, or other continuous renal replacement therapies), with a single evaluation by a physician or other qualified health care professional
	Demographics	Deceased	Deceased
**Respiratory infection**	Diagnosis	UMLS:ICD10CM:J18	Pneumonia, unspecified organism
	Diagnosis	UMLS:ICD10CM:J12	Viral pneumonia, not elsewhere classified
	Diagnosis	UMLS:ICD10CM:J20	Acute bronchitis
**Ketoacidosis**	Diagnosis	UMLS:ICD10CM:E11.10	Type 2 diabetes mellitus with ketoacidosis without coma
	Diagnosis	UMLS:ICD10CM:E11.11	Type 2 diabetes mellitus with ketoacidosis with coma
**Aortic aneurysm or dissection**	Diagnosis	UMLS:ICD10CM:I71	Aortic aneurysm and dissection
**Otitis media**	Diagnosis	UMLS:ICD10CM:H65	Nonsuppurative otitis media
	Diagnosis	UMLS:ICD10CM:H66	Suppurative and unspecified otitis media
	Diagnosis	UMLS:ICD10CM:H67	Otitis media in diseases classified elsewhere

3. Baseline parameters

Propensity score matching included all listed characteristics. The covariates and laboratory data missingness rates after matching are summarized below.

Covariates used for propensity score matching	
**Demographics**	
AI	Age at index
Male	Male
2106-3	White
2054-5	African American
2028-9	Asian
2131-1	Other race
**Lifestyles**	
ICD-10-CM code	Name
Z55-Z65	Persons with potential health hazards related to socioeconomic and psychosocial circumstances
Z59	Problems related to housing and economic circumstances
Z56	Problems related to employment and unemployment
Z72.0	Tobacco use
Z87.891	Personal history of nicotine dependence
F10	Alcohol-related disorders
**Diagnosis**	
ICD-10-CM code	Name
E78	Disorders of lipoprotein metabolism and other lipidemias
I10-I1A	Hypertensive diseases
I50	Heart failure
I48	Atrial fibrillation and flutter
I20-I25	Ischemic heart diseases
I73.9	Peripheral vascular disease, unspecified
N17-N19	Acute kidney failure and chronic kidney disease
I60-I69	Cerebrovascular diseases
J44	Other chronic obstructive pulmonary disease
M10	Gout
K74	Fibrosis and cirrhosis of liver

Covariates used for propensity score matching				
G00-G09	Inflammatory diseases of the central nervous system			
F01-F99	Mental, behavioral, and neurodevelopmental disorders			
**Medication**				
ATC Code	Name			
A10A	Insulins and analogues			
C10	Lipid-modifying agents			
C09	Agents acting on the renin-angiotensin system			
C03	Diuretics			
C07	Beta-blocking agents			
C08	Calcium channel blockers			
L	Antineoplastic and immunomodulating agents			
N03	Antiepileptics			
A10BB	Sulfonylureas			
A10BG	Thiazolidinediones			
A10BF	Alpha-glucosidase inhibitors			
A10BA	Biguanides			
A10BK	Sodium-glucose cotransporter 2 (SGLT2) inhibitors			
R06	Antihistamines for systemic use			
C01BD01	Amiodarone			
G03C	Estrogens			
**Laboratory**				
TNX-curated	Name	Unit	Missingness rate after PSM	
			GLP-1 RA users	DPP-4i users
9044	Alanine aminotransferase	U/L	51.41%	51.71%
9047	Aspartate aminotransferase	U/L	51.69%	51.95%
9004	Triglycerides	mg/dL	61.75%	61.92%
9083	BMI	kg/m^2	43.05%	42.24%
9037	Hemoglobin A1c	%	50.79%	51.85%
9002	Low-density lipoprotein cholesterol	mg/dL	62.76%	62.94%
9001	High-density lipoprotein cholesterol	mg/dL	61.46%	61.65%
9014	Hemoglobin	g/dL	54.71%	54.09%

**Covariates used for propensity score matching**				
9045	Albumin	g/dL	52.76%	52.71%
8001	Estimated glomerular filtration rate (MDRD)	mL/min/ 1.73 m²	45.23%	44.96%
9029	Sodium	mmol/L	44.10%	44.45%
9028	Potassium	mmol/L	44.65%	45.05%
9022	Calcium	mg/dL	45.05%	45.08%
9027	Phosphate	mg/dL	90.90%	90.89%
14959-1	Urine microalbumin-to-creatinine ratio	mg/g	88.04%	88.19%
9085	Systolic blood pressure	mmHg	41.73%	41.49%
9086	Diastolic blood pressure	mmHg	41.74%	41.51%

Method S2. Details of propensity score matching on the TriNetX platform (https://support.trinetx.com/hc/en-us/articles/360011978033)

To conduct PSM using the TriNetX Research platform, you must first identify two cohorts of interest, index events, outcomes of interest, and patient attributes that may act as confounders of the outcomes. For this article, these attributes are termed "covariates."

Within Balance, when you run a propensity score matching analysis, the system conducts propensity score matching to balance the cohorts:

1. For each patient in each cohort, the system computes values for each covariate.
2. The resulting data form a matrix of covariate values for each cohort.
3. The system performs logistic regression on the pooled matrices to predict the cohort from which each patient originates. The model output for a patient is that patient's predicted probability of belonging to the second cohort, or "propensity score."
4. For each patient in the smaller cohort, the system chooses a match from the larger cohort if an unmatched patient in the larger cohort is sufficiently close. The matched pairs then form a subset of each cohort.

Within Outcomes, when you compare cohorts after matching, the system conducts propensity score matching before comparing outcomes between the cohorts:

1. For each patient in each cohort, the system computes both the outcome(s) of interest and values for each covariate.
2. The resulting data form a matrix of covariate values for each cohort.
3. The system performs logistic regression on the pooled matrices to predict the cohort from which each patient originates. The model output for a patient is that patient's predicted probability of belonging to the second cohort, or "propensity score."
4. For each patient in the smaller cohort, the system chooses a match from the larger cohort if an unmatched patient in the larger cohort is sufficiently close. The matched pairs then form a subset of each cohort.
5. The system compares outcomes in these matched subsets rather than in the original cohorts.

Values in the covariate matrix

In the covariate matrix, each row represents one patient and each column represents one covariate. Each cell contains exactly one non-null numerical value.

All covariates take one of the following forms:

- **Binary:** yes or no;
- **Categorical:** continuous values grouped into categories based on their values; or
- **Continuous:** real values represented directly.

When a covariate is binary (for example, a patient with a history of asthma), the cell for that patient and covariate is set to 0 for "not present" or 1 for "present."

When a covariate is categorical, categories are specified as ranges of possible continuous values (for example, a sodium value between 130 and 140). Each category becomes a distinct covariate coded 0 for "not present" or 1 for "present." All columns may be 0 if no value is recorded within the time window or if all values are outside the specified ranges. Multiple columns may be 1 if multiple values occur within the time window or if categories overlap.

When a covariate is continuous (for example, current age or age at the index event), the patient's value, if available, is placed in the corresponding cell.

Propensity score estimation and logistic regression

When propensity score matching is run, the system generates a propensity score for each patient in each cohort. The score ranges from 0 to 1 and represents the predicted probability that the patient belongs to cohort B given the patient's covariates.

The system uses logistic regression, implemented in the well-tested scikit-learn software package, to generate propensity scores. The production code is shown below, with logging and other nonessential elements omitted for clarity:

```
import numpy as np
from sklearn.linear_model import LogisticRegression
from sklearn.preprocessing import Imputer
from impute import fill_nans
def fill_nans(data, axis=0):
    imp = Imputer(missing_values='NaN', strategy=method, axis=axis)
    return imp.fit_transform(data)
def propensity_scores(matrix_a, matrix_b):
    matrix_a = fill_nans(matrix_a, method='mean')
    matrix_b = fill_nans(matrix_b, method='mean')
    full_matrix = np.concatenate((matrix_a, matrix_b), axis=0)
    # cohort A is "zero" and cohort B is "one" from the regression’s perspective
    target_a = np.zeros(matrix_a.shape[0], dtype=float)
    target_b = np.ones(matrix_b.shape[0], dtype=float)
```

```
    target = np.concatenate([target_a, target_b])
    lr = LogisticRegression(C=1000)
    lr.fit(full_matrix, target)
    def get_scores(X):
        # .predict_proba returns [prob_class_zero, prob_class_one] for each row,
        # while the score we want is the probability of being in class B
        full_scores = lr.predict_proba(X)
        return full_scores[:, 1]
    return (get_scores(matrix_a), get_scores(matrix_b))
```

For each patient, the "score" is the regression model applied to that patient's row. This score is the estimated probability that the patient belongs to cohort B based on the observed covariates.

Here, "matrix_a" is the covariate matrix for cohort A, and "matrix_b" is the covariate matrix for cohort B. Data are pooled across all HCOs, so each patient contributes a row to one of these matrices. The code replaces missing (NaN) values in each matrix with the mean of the corresponding column; however, at the time of writing, all covariates were binary, categorical, or continuous values required to be present, so this imputation was unnecessary.

The call "LogisticRegression(C=1000)" predicts the probability of belonging to cohort B using logistic regression. The value of C is the inverse of regularization strength, so C=1000 applies only a very small amount of L2 regularization. This weak regularization supports convergence but has little effect on the fitted model. Because covariate scales are constrained, mean normalization is not required as a preprocessing step.

Matching procedure

After the system has generated a propensity score for each patient, it performs greedy nearest-neighbor matching with a caliper of 0.1 pooled standard deviations to identify matched subsets. Sample code is shown below:

```
import numpy as np
def _pooled_sd(floats_a, floats_b):
    var_a = np.var(floats_a)
    var_b = np.var(floats_b)
    return sqrt((var_a + var_b) / 2.0)
def _naive_nearest_neighbor_match(scores_a, scores_b, caliper):
```

```
# Perform nearest neighbor matching. Assume that len(scores_a) <=len(scores_b)
num_a, num_b = len(scores_a), len(scores_b)
max_diff = _pooled_sd(scores_a, scores_b) * caliper
chosen_a = np.zeros(num_a, dtype=bool)  # True if the patient is matched, start false
chosen_b = np.zeros(num_b, dtype=bool)
for a_ind in range(0, num_a):
    best_diff = max_diff
    best_b_ind = None
    found_any = False
    for b_ind in range(0, num_b):
        if chosen_b[b_ind]:
            continue
        diff = abs(scores_a[a_ind] - scores_b[b_ind])
        if diff < best_diff or (diff == best_diff and not found_any):
            best_diff = diff
            best_b_ind = b_ind
            found_any = True
    if best_b_ind is not None:
        chosen_a[a_ind] = True
        chosen_b[best_b_ind] = True
return (chosen_a, chosen_b)
```

The production code is highly optimized and therefore substantially longer. The simplified code above is taken from test fixtures used to ensure that the optimized code produces identical results.

A caliper of 0.1 pooled standard deviations of the propensity scores is used, so patients with very different propensity scores are not matched. The system applies the following algorithm:

1. For each patient in cohort A (assumed to be the smaller cohort), identify the unmatched patient in cohort B whose score is closest to that of the patient in cohort A.
2. If a match is found, mark both patients as "chosen" and proceed to the next patient.
3. At the end, return the cohort labels indicating which patients were chosen. The chosen patients form the matched cohorts.

Pooling cohorts and addressing imbalance across HCOs

TriNetX draws data from a federated network of healthcare organizations worldwide. Each site computes a covariate matrix for the patients it contributes and sends the matrix to a central processing point to be pooled and analyzed as a single matrix.

The order of rows in the matrix should not affect the propensity scores generated for each patient because logistic regression is highly stable with respect to row permutations. In contrast, nearest-neighbor matching can be influenced by row order. For example, if two identical patients in cohort B are equally suitable matches for a patient in cohort A, the first will be chosen and the second will not. Therefore, if row order carries information as an artifact of pooling, it can introduce bias.

To reduce this bias, we randomize the order of records in each covariate matrix. We sort provider results by the unique HCO identifier, thereby removing information about which HCO responded first, concatenate the matrices, and then shuffle them using `np.random.shuffle(full_matrix_a)` and `np.random.shuffle(full_matrix_b)`. To ensure determinism, a call to `np.random.seed(FIXED_SEED)` precedes all shuffling so that successive runs do not change unless the underlying data change.

Figure S1. Love plot

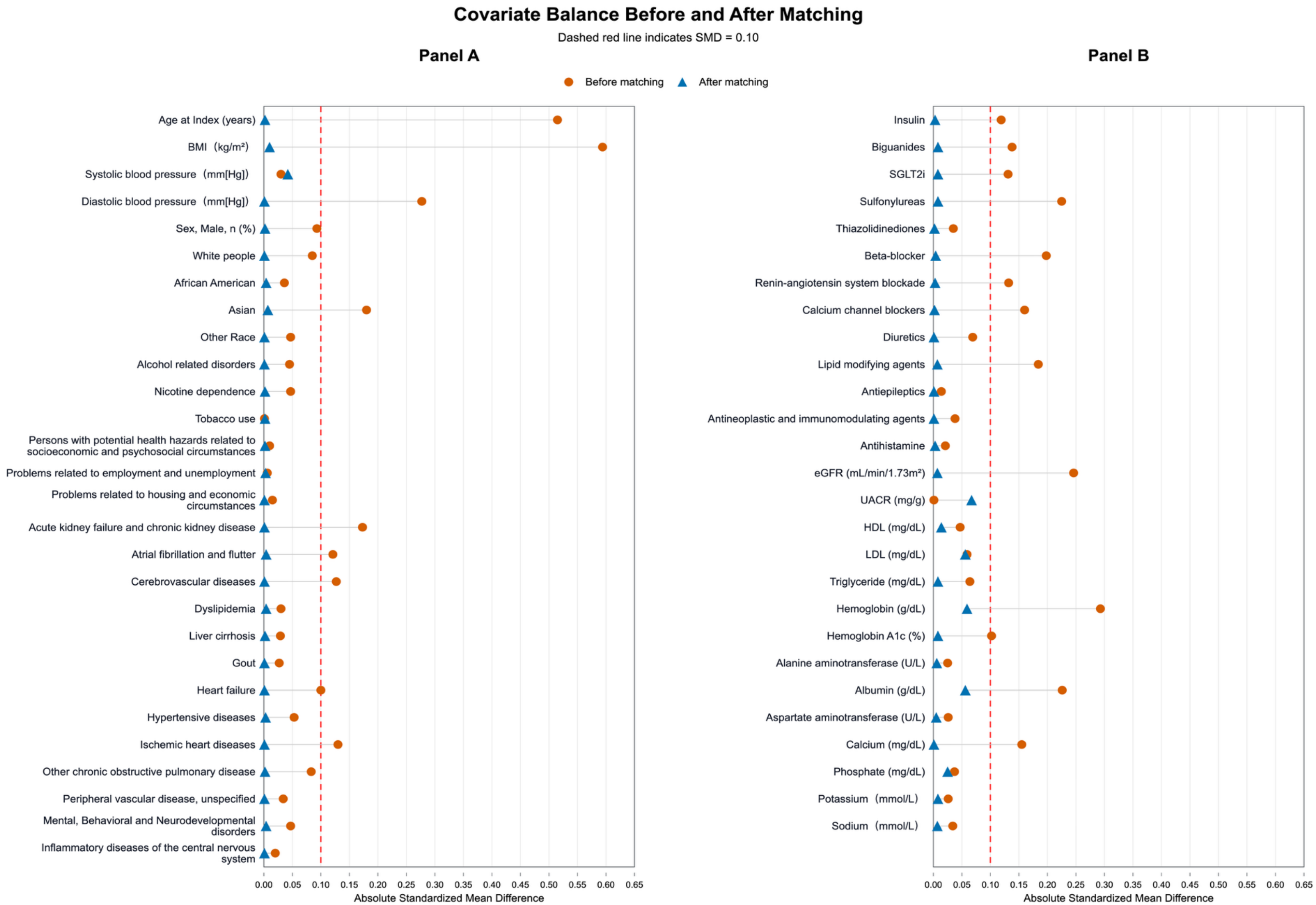

Figure S2. Distribution of follow-up time

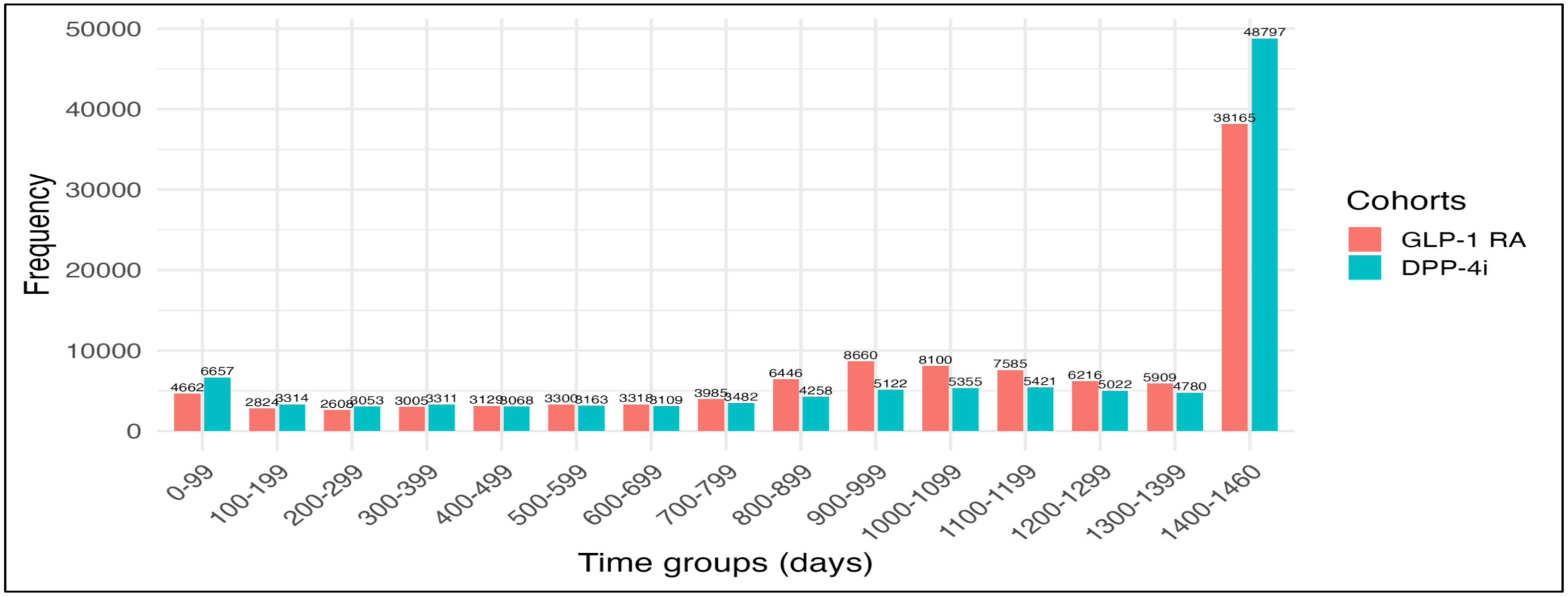

**After Matching Follow-up Time Results**					
Cohort	Cohort Name	Mean Follow-up (Days)	Standard Deviation	Median Follow-up (Days)	Interquartile Range
1	GLP-1 RA	1043.5	438.6	1144	662
2	DPP-4i	1059.4	481.9	1287	726

Figure S3. Kaplan–Meier plots for secondary outcomes

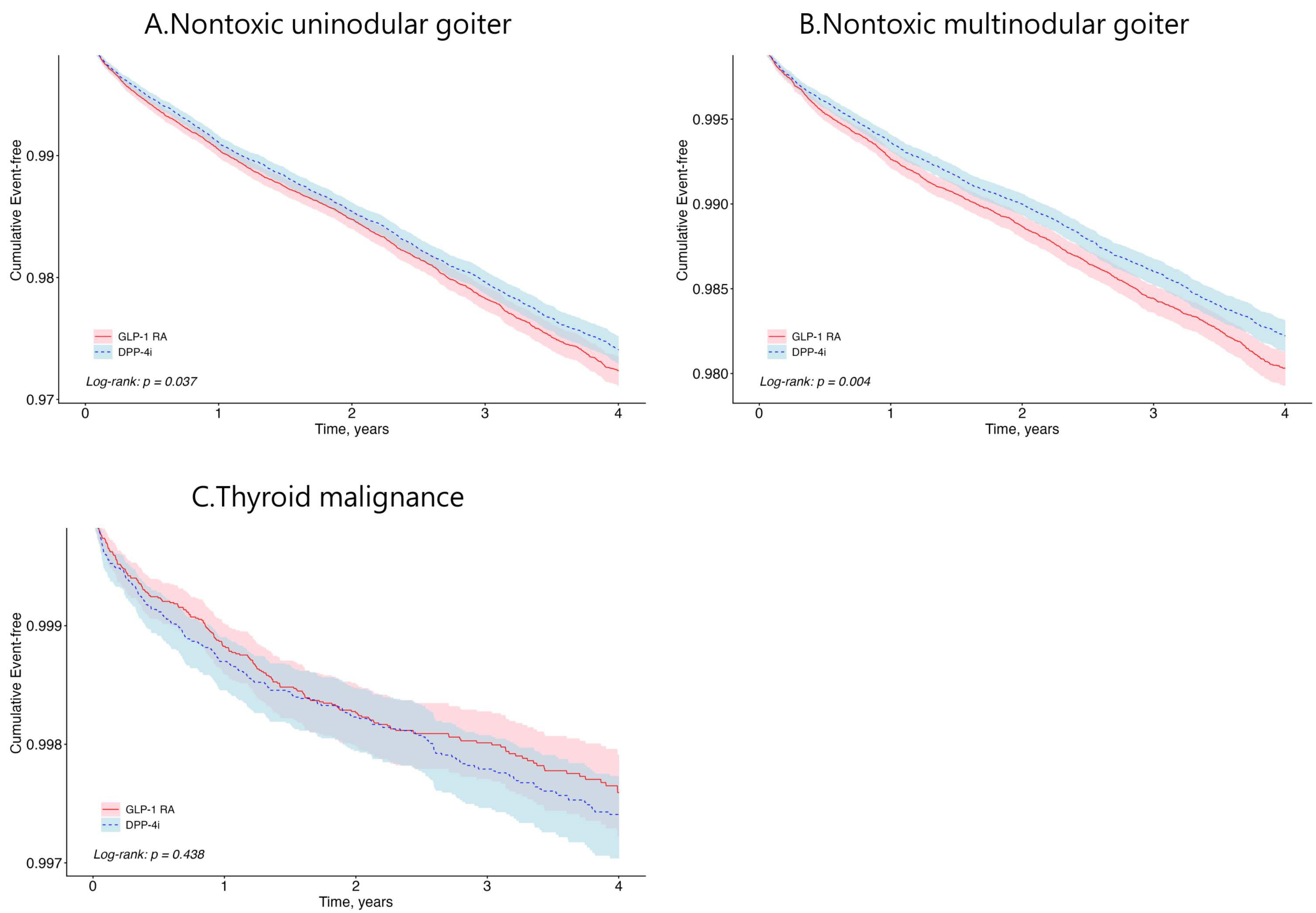

Table S1. Target trial emulation

Target trial protocol component	Ideal randomized controlled trial	Emulation in our observational study
**Research question**	Among adults with type 2 diabetes mellitus who require incretin-based therapy, does initiating a GLP-1 receptor agonist increase the risk of autoimmune thyroiditis compared with initiating a DPP-4 inhibitor?	We estimated the association between GLP-1 RA initiation and the risk of autoimmune thyroiditis compared with DPP-4i initiation among adults with T2D in the TriNetX US Collaborative Network.
**Eligibility criteria**	Adults aged ≥18 years with diagnosed T2D who were eligible to initiate either GLP-1 RA or DPP-4i and had no prior exposure to either drug class or history of thyroid disease, any neoplasm, immunosuppressant or immunostimulant therapy, transplant status, or end-stage kidney disease or dialysis.	Adults aged ≥18 years with T2D between January 1, 2018, and December 31, 2022, who had at least three clinical encounters. Patients with prior GLP-1 RA or DPP-4i use, thyroid disorders, immunosuppressant or immunostimulant use, any neoplasm, transplant status, or end-stage kidney disease or dialysis were excluded.
**Treatment strategies**	Strategy 1: initiate GLP-1 RA. Strategy 2: initiate DPP-4i. Participants would remain assigned to their randomized treatment strategy regardless of discontinuation, switching, or add-on therapy, consistent with the intention-to-treat principle.	Strategy 1: initiate GLP-1 RA as a new user. Strategy 2: initiate DPP-4i as a new user. The study used a new-user, active-comparator, intention-to-treat design.
**Assignment procedure**	Eligible participants would be randomly assigned 1:1 to initiate GLP-1 RA or DPP-4i, ensuring exchangeability of measured and unmeasured baseline characteristics.	Treatment was not randomized. To emulate randomization, we used 1:1 propensity score matching with nearest-neighbor matching without replacement to balance measured baseline covariates between GLP-1 RA and DPP-4i initiators.
**Index date and time zero**	Date of randomization and treatment initiation. Eligibility criteria, treatment assignment, and start of follow-up would occur at the same time.	The index date was the date of the first prescription for GLP-1 RA or DPP-4i. Baseline covariates were assessed during the 2 years preceding the index date.
**Baseline covariates**	Baseline demographic, clinical, medication, lifestyle, socioeconomic, and laboratory variables would be measured before randomization.	Baseline covariates included age, sex, race, BMI, blood pressure, lifestyle factors, socioeconomic variables, comorbidities, medications, and laboratory values, including eGFR, HbA1c, lipids, hemoglobin, liver enzymes, albumin, calcium, phosphate, potassium, and sodium.

Table S1. Target trial emulation

Target trial protocol component	Ideal randomized controlled trial	Emulation in our observational study
**Primary outcome**	Incident autoimmune thyroiditis after treatment initiation, ideally confirmed by clinical diagnosis, thyroid autoantibodies, thyroid function tests, and/or physician adjudication.	The primary outcome was incident autoimmune thyroiditis identified using diagnostic codes in TriNetX after the index date.
**Secondary outcomes**	Incident nontoxic uninodular goiter, nontoxic multinodular goiter, and thyroid malignancy, assessed using standardized clinical criteria.	Secondary outcomes included nontoxic uninodular goiter, nontoxic multinodular goiter, and malignant neoplasm of the thyroid gland, identified using diagnostic codes.
**Follow-up period**	Participants would be followed from randomization until a thyroid outcome, death, loss to follow-up, trial end, or a maximum follow-up of 4 years.	Patients were followed for outcomes from 1 day after the index date through 4 years; observations extended through June 20, 2025.
**Causal contrast of interest**	The intention-to-treat effect of initiating GLP-1 RA versus DPP-4i on the risk of autoimmune thyroiditis. A per-protocol effect could also be estimated if adherence and switching were fully measured.	The primary estimand was the observational analogue of the intention-to-treat effect comparing initiation of GLP-1 RA versus DPP-4i after propensity score matching.
**Statistical analysis**	Cumulative incidence would be compared, and hazard ratios would be estimated using survival models while preserving randomized groups. Prespecified subgroup and sensitivity analyses would be performed.	Kaplan–Meier curves, log-rank tests, and Cox proportional hazards models were used to estimate hazard ratios and 95% confidence intervals. Proportional hazards assumptions were assessed using the generalized Schoenfeld approach in TriNetX.
**Confounding control**	Randomization would control for both measured and unmeasured confounding.	Propensity score matching was used to control for measured baseline confounders. Balance was assessed using standardized mean differences, with SMD < 0.1 indicating acceptable balance. Residual confounding from unmeasured factors remains possible.
**Sensitivity analyses**	Robustness would be assessed under alternative lag periods, adherence definitions, censoring assumptions, and outcome definitions. Positive and negative control outcomes would be included if feasible.	Sensitivity analyses included a 3-month lag window, multiple propensity score matching models with stepwise adjustment, different follow-up durations, and positive and negative control outcomes. Positive controls included MACE, MAKE, and respiratory infection; negative controls included ketoacidosis, aortic aneurysm or dissection, and otitis media.

Table S1. Target trial emulation

Target trial protocol component	Ideal randomized controlled trial	Emulation in our observational study
**Subgroup analyses**	Prespecified subgroups could include age, sex, race, BMI, renal function, HbA1c, nicotine dependence, and major comorbidities.	Subgroup analyses were performed by sex, age, race, BMI, eGFR, HbA1c, nicotine dependence, hypertension, heart failure, ischemic heart disease, chronic kidney disease, dyslipidemia, and mental disorders.
**Main expected advantage**	High internal validity due to randomization and standardized outcome assessment.	Greater feasibility, larger sample size, greater real-world generalizability, and the ability to study uncommon outcomes such as autoimmune thyroiditis using routinely collected data.
**Main limitation**	High cost, long duration, and potentially lengthy follow-up; rare thyroid outcomes may require a very large sample.	Nonrandom treatment allocation, possible residual confounding, outcome misclassification based on diagnostic codes, inability to measure variables such as TPO antibody titers, iodine status, thyroid ultrasound findings, or family history, and possible surveillance bias.

Abbreviations: BMI, body mass index; CI, confidence interval; CKD, chronic kidney disease; DPP-4i, dipeptidyl peptidase-4 inhibitor; eGFR, estimated glomerular filtration rate; GLP-1 RA, glucagon-like peptide-1 receptor agonist; HbA1c, hemoglobin A1c; HR, hazard ratio; ITT, intention-to-treat; MACE, major adverse cardiovascular event; MAKE, major adverse kidney event; PSM, propensity score matching; RCT, randomized controlled trial; SMD, standardized mean difference; T2D, type 2 diabetes.

Table S2. Annual absolute risk metrics and clinical impact measures

Outcome/metric	GLP-1 RA versus DPP-4i			
	Year 1	Year 2	Year 3	Year 4
**Autoimmune thyroiditis**				
[a]ARI, % (95% CI)	0.04 (-0.01, 0.08)	0.09 (0.03, 0.14)	0.09 (0.03, 0.16)	0.14 (0.06, 0.21)
[b]NNH	2394	1162	1087	733

[a] ARI, absolute risk increase; positive values indicate higher risk with GLP-1 RA than with DPP-4i.
[b] NNH, number needed to harm, calculated as 1/ARI with ARI expressed as a proportion.
Estimates are from the Kaplan–Meier cumulative incidence at the end of each year in the matched cohorts of the primary analysis (107,912 patients per group). Abbreviations: CI, confidence interval; DPP-4i, dipeptidyl peptidase-4 inhibitor; GLP-1 RA, glucagon-like peptide-1 receptor agonist.

Table S3. Positive and negative control outcomes after matching

Clinical outcomes	GLP-1 RA (n = 106,383)		DPP-4i (n = 106,383)		GLP-1 RA versus DPP-4i	
	Events	Risk (%)	Events	Risk (%)	HR (95% CI)	*p-value*
**Positive control**						
MACE	13,769	12.9	15,445	14.5	0.90 (0.87–0.92)	<0.001
MAKE	15,683	14.7	18,474	17.4	0.85 (0.83–0.86)	<0.001
Respiratory infection	8,855	8.3	10,115	9.5	0.88 (0.85–0.90)	<0.001
**Negative control**						
Ketoacidosis	1,516	1.4	1,592	1.5	0.96 (0.90–1.03)	0.302
Aortic aneurysm or dissection	1,573	1.5	1,585	1.5	0.99 (0.93–1.07)	0.926
Otitis media	2,628	2.5	2,538	2.4	1.05 (0.99–1.11)	0.105

Risk is the proportion of patients in each matched cohort with the outcome during 4 years of follow-up. Hazard ratios are from Cox proportional hazards models and P values from log-rank tests. Control outcomes were defined with the codes listed in Method S1. Abbreviations: CI, confidence interval; DPP-4i, dipeptidyl peptidase-4 inhibitor; GLP-1 RA, glucagon-like peptide-1 receptor agonist; HR, hazard ratio; MACE, major adverse cardiovascular events (death, acute myocardial infarction, cerebral infarction, other nontraumatic intracranial hemorrhage, and cardiac arrest); MAKE, major adverse kidney events (acute kidney failure, end-stage renal disease, dialysis services and procedures, dialysis procedures other than hemodialysis, and death).

Table S4. Models for confounding adjustment after matching

Clinical outcomes	GLP-1 RA versus DPP-4i users					
	Model 1		Model 2		Model 3	
	HR (95% CI)	*p*-value	HR (95% CI)	*p*-value	HR (95% CI)	*p*-value
**Primary outcome**						
Autoimmune thyroiditis	1.26 (1.09–1.46)	0.001	1.31 (1.16–1.48)	<0.001	1.21 (1.07–1.38)	0.003
**Secondary outcomes**						
Nontoxic uninodular goiter	1.06 (0.99–1.13)	0.075	1.08 (1.02–1.14)	0.012	1.07 (1.01–1.13)	0.025
Nontoxic multinodular goiter	1.09 (1.01–1.17)	0.037	1.13 (1.06–1.21)	<0.001	1.11 (1.03–1.19)	0.005
Malignant neoplasm of thyroid gland	0.78 (0.63–0.96)	0.016	0.87 (0.73–1.05)	0.143	0.83 (0.69–0.99)	0.049

Each model repeated the propensity score matching with a different covariate set and compared GLP-1 RA with DPP-4i over 4 years of follow-up. Model 1 matched on age, sex, race, lifestyle factors, body mass index, blood pressure, and economic circumstances; Model 2 added baseline comorbidities; Model 3 added baseline medication use. Matching produced 102,492, 116,672, and 109,993 pairs per group for Models 1, 2, and 3, respectively. Hazard ratios are from Cox proportional hazards models and P values from log-rank tests.
Abbreviations: CI, confidence interval; DPP-4i, dipeptidyl peptidase-4 inhibitor; GLP-1 RA, glucagon-like peptide-1 receptor agonist; HR, hazard ratio.

Table S5. Sensitivity analysis by follow-up duration after matching

Clinical outcomes	GLP-1 RA versus DPP-4i users					
	1 day to 1 year		1 day to 2 years		1 day to 3 years	
	HR (95% CI)	*p-value*	HR (95% CI)	*p-value*	HR (95% CI)	*p-value*
**Primary outcome**						
Autoimmune thyroiditis	1.21 (1.01–1.46)	0.038	1.24 (1.06–1.45)	0.008	1.15 (0.98–1.34)	0.081
**Secondary outcomes**						
Nontoxic uninodular goiter	0.98 (0.89–1.08)	0.707	0.99 (0.92–1.08)	0.973	1.03 (0.96–1.11)	0.357
Nontoxic multinodular goiter	1.18 (1.06–1.32)	0.003	1.14 (1.04–1.25)	0.005	1.10 (1.01–1.20)	0.031
Malignant neoplasm of thyroid gland	0.90 (0.70–1.17)	0.431	0.86 (0.69–1.08)	0.199	0.97 (0.78–1.20)	0.751

Each follow-up window was analyzed as a separate propensity score-matched analysis with the same covariates as the primary analysis; matching produced 101,026, 100,351, and 95,831 pairs per group for the 1-, 2-, and 3-year windows, respectively. Results for the full 4-year window are shown in Table 2. Hazard ratios are from Cox proportional hazards models and P values from log-rank tests. Abbreviations: CI, confidence interval; DPP-4i, dipeptidyl peptidase-4 inhibitor; GLP-1 RA, glucagon-like peptide-1 receptor agonist; HR, hazard ratio.

Table S6. Sensitivity analysis using a 3-month exposure lag window

Clinical outcomes	GLP-1 RA (n = 98,319)		DPP-4i (n = 98,319)		GLP-1 RA versus DPP-4i	
	**Events**	**Cumulative incidence risk (%)**	**Events**	**Cumulative incidence risk (%)**	**HR (95% CI)**	***p-value***
**Primary outcome**						
Autoimmune thyroiditis	306	0.31	252	0.26	1.23 (1.04–1.46)	0.014
**Secondary outcomes**						
Nontoxic uninodular goiter	1,594	1.63	1,513	1.54	1.08 (1.01–1.16)	0.030
Nontoxic multinodular goiter	1,010	1.03	988	1.01	1.05 (0.97–1.15)	0.237
Malignant neoplasm of thyroid gland	112	0.11	121	0.12	0.93 (0.72–1.21)	0.606

Outcomes were counted from day 90 to day 1550 after the index date, that is, 4 years of follow-up after a 3-month exposure lag. Patients with the outcome before the start of that window were excluded from the analysis of that outcome, so the number at risk is slightly smaller than the matched cohort size shown in the column headings. Cumulative incidence risk is the proportion of patients at risk with the outcome; hazard ratios are from Cox proportional hazards models and P values from log-rank tests. Abbreviations: CI, confidence interval; DPP-4i, dipeptidyl peptidase-4 inhibitor; GLP-1 RA, glucagon-like peptide-1 receptor agonist; HR, hazard ratio.

Table S7. Thyroid testing, thyroid ultrasound, outpatient visits, and thyroid diagnoses after treatment initiation in the matched cohorts

Measure	Within 1 year after the index date			Within 4 years after the index date		
	**GLP-1 RA (n = 116,614)**	**DPP-4i (n = 116,614)**	**SMD**	**GLP-1 RA (n = 129,956)**	**DPP-4i (n = 129,956)**	**SMD**
TSH testing, n (%)	17,297 (14.8)	14,510 (12.4)	0.070	39,125 (30.1)	32,975 (25.4)	0.106
TSH tests per tested patient, mean ± SD	1.33 ± 0.78	1.34 ± 0.81	0.015	2.46 ± 2.15	2.45 ± 2.18	0.006
Free T4 testing, n (%)	8,539 (7.3)	8,422 (7.2)	0.004	21,219 (16.3)	20,556 (15.8)	0.014
Thyroid or neck ultrasound, n (%)	1,240 (1.1)	1,016 (0.9)	0.020	3,935 (3.0)	3,359 (2.6)	0.027
Any outpatient visit, n (%)	63,223 (54.2)	59,205 (50.8)	0.069	75,102 (57.8)	73,074 (56.2)	0.032
Visits per patient with visits, mean ± SD	4.61 ± 4.46	4.20 ± 4.14	0.096	13.71 ± 14.10	11.94 ± 12.50	0.133
**Thyroid diagnosis**	**GLP-1 RA n (%)**	**DPP-4i n (%)**	**HR (95% CI)**	**GLP-1 RA n (%)**	**DPP-4i n (%)**	**HR (95% CI)**
Any thyroid disorder	7,281 (6.2)	7,014 (6.0)	1.02 (0.99–1.06)	14,812 (11.4)	14,216 (10.9)	1.02 (0.99–1.05)

The two time windows were analyzed separately in TriNetX using the same propensity score model as the primary analysis; matched cohort sizes therefore differ from those in Table 2. TSH testing was identified with LOINC 3016-3, free T4 testing with LOINC 3024-7, thyroid or neck ultrasound with CPT 76536, outpatient visits with CPT office or other outpatient services codes, and thyroid disorders with ICD-10-CM E00–E07, which excluded patients from both cohorts when recorded before the index date. Tests were counted by date and visits by encounter; mean counts are among patients with at least one instance. An SMD below 0.1 indicates a negligible difference between the groups. Hazard ratios for any thyroid disorder were estimated with Cox proportional hazards models for the first recorded diagnosis in each window, with censoring after the last recorded clinical event. Abbreviations: CI, confidence interval; DPP-4i, dipeptidyl peptidase-4 inhibitor; GLP-1 RA, glucagon-like peptide-1 receptor agonist; HR, hazard ratio; SD, standard deviation; SMD, standardized mean difference; T4, thyroxine; TSH, thyroid-stimulating hormone.

Table S8. Extra-thyroidal autoimmune diseases before propensity score matching and bias analysis for autoimmune thyroiditis

Condition (ICD-10-CM code)	GLP-1 RA (n = 240,286)	DPP-4i (n = 145,491)	SMD
Type 1 diabetes mellitus (E10)	5,582 (2.32)	1,775 (1.22)	0.084
Rheumatoid arthritis with rheumatoid factor (M05)	293 (0.12)	183 (0.13)	0.001
Other rheumatoid arthritis (M06)	1,228 (0.51)	922 (0.63)	0.016
Systemic lupus erythematosus (M32)	405 (0.17)	225 (0.15)	0.003
Sjögren syndrome (M35.0)	246 (0.10)	130 (0.09)	0.004
Systemic sclerosis (M34)	53 (0.02)	43 (0.03)	0.005
Psoriasis (L40)	1,852 (0.77)	791 (0.54)	0.028
Vitiligo (L80)	96 (0.04)	36 (0.02)	0.008
Alopecia areata (L63)	69 (0.03)	18 (0.01)	0.011
Crohn's disease (K50)	350 (0.15)	215 (0.15)	0.001
Ulcerative colitis (K51)	353 (0.15)	311 (0.21)	0.016
Celiac disease (K90.0)	190 (0.08)	71 (0.05)	0.012
Multiple sclerosis (G35)	343 (0.14)	223 (0.15)	0.003
Pernicious anemia (D51.0)	159 (0.07)	97 (0.07)	<0.001
**Assumed HR of the conditions for autoimmune thyroiditis**	**2**	**3**	**5**
Corrected HR (95% CI), GLP-1 RA versus DPP-4i	1.24 (1.09–1.41)	1.23 (1.08–1.40)	1.21 (1.06–1.37)

Table S8. Extra-thyroidal autoimmune diseases before propensity score matching and bias analysis for autoimmune thyroiditis

Condition (ICD-10-CM code)	GLP-1 RA (n = 240,286)	DPP-4i (n = 145,491)	SMD

Values are n (%). Conditions were identified by ICD-10-CM codes recorded in the 2 years before the index date, from a separate TriNetX query before propensity score matching (cohort sizes differ from Table 1); percentages use the total cohort sizes as denominators. An SMD below 0.1 indicates a negligible difference between the groups. Corrected HRs were calculated from the unrounded primary HR (1.26; 95% CI 1.11–1.43) with the formula of Lin et al. (Biometrics. 1998; 54: 948–63) for a binary unmeasured confounder, treating the 14 conditions as one confounder with the summed prevalence (4.67% and 3.46%, calculated from the counts; overlap ignored).
Abbreviations: CI, confidence interval; DPP-4i, dipeptidyl peptidase-4 inhibitor; GLP-1 RA, glucagon-like peptide-1 receptor agonist; HR, hazard ratio; ICD-10-CM, International Classification of Diseases, Tenth Revision, Clinical Modification; SMD, standardized mean difference.
